# Supplementary material for: Effects of Exergaming in Patients with Cardiovascular Disease Compared to Conventional Cardiac Rehabilitation: A Systematic Review and Meta-Analysis
Source: Int J Environ Res Public Health. 2022 Mar 15;19(6):3492. doi: 10.3390/ijerph19063492 (PMC8950475; doi:10.3390/ijerph19063492)
Supplement: Supplementary file 1 [file ijerph-19-03492-s001.zip › ijerph-1613586-supplementary.pdf]

International Journal of
Environmental Research and Public Health

MDPI

Supplementary Material

Electronic database search.

(1) Patients: “heart fail*” OR “cardiac fail*” OR “failing heart*” OR “myocardial insufficiency” OR “myocardial fail*” OR “failing myocard*” OR “failure myocard*” OR “heart decompensation” OR “impaired myocardial function” OR “reduced myocardial function” OR HFrEF OR HFpEF OR hypertension OR “myocardial infarction” OR “ischemic heart disease” OR “cardiovascular disease*” OR CVD OR “coronary heart disease” OR ″coronary artery disease” OR “coronary artery bypass grafting” OR “acute coronary syndrome” OR “percutaneous coronary intervention” OR atherosclerosis OR “angina pectoris” OR “heart disease*” OR “cardiac rehab*”
(2) Interventions: game* OR videogame* OR exergam* OR gami* OR "virtual reality" OR "virtual rehab*" OR "augmented reality" OR ludic OR Wii OR Kinect OR “virtual environment*” OR “virtual world*” OR “virtual therap*”
(3) #1 AND #2
